# Parosmia in patients with post-infectious olfactory dysfunction in the era of COVID-19-associated olfactory impairment

**DOI:** 10.1007/s00106-024-01470-7

**Published:** 2024-06-27

**Authors:** Nadine Gunder, Thomas Hummel

**Affiliations:** Department of Otolaryngology, Head and Neck Surgery, Faculty of Medicine and University Hospital Carl Gustav Carus, TU Dresden, Fetscherstraße 74, 01307 Dresden, Germany

**Keywords:** Smell, Olfaction, Olfactory dysfunction, Parosmia, COVID-19, Geruch, Geruchssinn, Riechstörung, Parosmie, COVID-19

## Abstract

**Objectives:**

A large number of patients with olfactory impairment are affected by parosmia or phantosmia. This study aimed to examine the demographic and clinical characteristics of parosmia.

**Methods:**

We performed a retrospective data analysis of patients consulting at our Smell and Taste Outpatient Clinic. A total of 297 patients were included (203 women, mean age 44.4 ± 13.7 years). Olfactory function was quantified using the “Sniffin’ Sticks” composite TDI (odor *t*hreshold, *d*etermination, and *i*dentification) score. The presence of qualitative olfactory impairment was assessed trough medical history and a parosmia questionnaire.

**Results:**

Most of the patients showed olfactory impairment after an infection with SARS-CoV‑2 (84%) and were diagnosed with parosmia (49%). Patients with parosmia (PAR) (*n* = 201) were significantly younger compared to the group without parosmia (noPAR; *n* = 92) (PAR 43.2 ± 13 years vs. noPAR 47 ± 15.1 years, *p* = 0.03) and had a slightly shorter duration of disease, without reaching statistical significance (PAR 10.3 ± 4.9 months, noPAR 13.6 ± 37.6 months, *p* = 0.23). They also had higher TDI scores (PAR 24.3 ± 7 points, noPAR 21.4 ± 8.2 points, *p* = 0.003).

**Conclusions:**

Patients affected by parosmia were younger and had a better olfactory function compared to patients without parosmia.

## Abstract (de)

**Zielsetzungen:**

Eine große Anzahl von Patient:innen mit Riechstörungen ist von Parosmie oder Phantosmie betroffen. Ziel dieser Studie war es, die demografischen und klinischen Merkmale der Parosmie zu untersuchen.

**Methoden:**

Die Autor:innen haben eine retrospektive Datenanalyse von Patient:innen durchgeführt, die in der Riech- und Geschmacksambulanz der Klinik der Autor:innen behandelt wurden. Insgesamt wurden 297 Patient:innen in die Studie eingeschlossen (203 Frauen, Durchschnittsalter 44,4 ± 13,7 Jahre). Die Riechfunktion wurde anhand des SDI-Werts (Schwellenwert, Diskrimination und Identifikation) der „Sniffin’ Sticks“ quantifiziert. Das Vorhandensein einer qualitativen Riechstörung wurde mittels der Krankengeschichte und eines Parosmie-Fragebogens ermittelt.

**Ergebnisse:**

Die meisten Patient:innen wiesen nach einer Infektion mit SARS-CoV‑2 eine Riechstörung auf (84 %), und bei ihnen wurde eine Parosmie diagnostiziert (49 %). Die Patient:innen mit Parosmie (PAR; *n* = 201) waren signifikant jünger als die Gruppe ohne Parosmie (noPAR; *n* = 92; PAR 43,2 ± 13 Jahre vs. noPAR 47 ± 15,1 Jahre; *p* = 0,03) und hatten eine etwas kürzere Krankheitsdauer, ohne dass dies statistisch signifikant war (PAR 10,3 ± 4,9 Monate; noPAR 13,6 ± 37,6 Monate; *p* = 0,23). Sie wiesen höhere SDI-Werte auf (PAR 24,3 ± 7 Punkte; noPAR 21,4 ± 8,2 Punkte; *p* = 0,003).

**Schlussfolgerungen:**

Die von Parosmie betroffenen Patient:innen waren jünger und hatten eine bessere Riechfunktion als Patient:innen ohne Parosmie.

## Introduction

Olfactory disorders are common with approximately one fifth of the population being affected [4, 65]. Due to the coronavirus pandemic, olfaction came to the attention of the general population because an infection with SARS-CoV‑2 is often associated with quantitative as well as qualitative olfactory impairment, such as parosmia and phantosmia [53]. Even before the pandemic, olfactory dysfunction (OD) following infections of the upper respiratory tract was one of the most common causes of olfactory loss [14, 63].

Qualitative OD is often, but not necessarily, accompanied by quantitative olfactory dysfunction [25]. Parosmia, meaning a distorted sensation of smell, is often associated with infections or head trauma [2, 9, 33, 66, 68]. Affected people describe the distorted smells mostly as unpleasant and disgusting, which affects their daily life. They report feeling distressed, anxious, and worried about their future [52], leading to mental health problems and in the most severe cases to clinical depression [12, 29]. Furthermore, they suffer from loss of appetite and reject certain foods because they are unable to tolerate many odors and flavors, resulting in reduced pleasure in food intake, changes in dietary patterns, social behaviors related to dining, and changes in weight [11, 16]. Olfactory impairment in general often results in reduced enjoyment of food [44].

Parosmia is thought to be a sign of recovery. However, the literature on this topic varies [24, 39, 56, 59]. Moreover, the pathogenesis of parosmia is widely debated. In the past, two different hypotheses have often been proposed. In “peripheral” theory, it is assumed that a loss of intact olfactory neurons results in the inability to form a complete “picture” of the odorant [17, 20, 37]; in “central” theory, the integration or interpretation in the brain is impaired and a distorted odor is formed [36]. The results of a recent study support the peripheral theory by demonstrating a decreased olfactory threshold in COVID-19 associated with OD [47].

Patients with SARS-CoV-2 rarely complain of additional rhinitis symptoms, and pathophysiological considerations suggest that the OD is due to damage to the olfactory mucosa [5, 64] rather than nasal obstruction/congestion [6]. SARS-CoV‑2 enters the cells via the surface receptor ACE2 [46], which is mainly expressed by the supporting cells in the olfactory mucosa. This is the most likely explanation for the rapid improvement in olfactory function in many cases, since the olfactory receptor neurons themselves are not directly affected [6]. A possible entry of the virus via Neuropilin 1 [7] and transport via the olfactory and/or trigeminal nerve into the brain is discussed [28]. However, the rapid recovery of symptoms in most cases contradicts this hypothesis [6].

In post-infectious OD (PIOD), improvement of olfactory function is most frequently compared with other causes of OD [66] and often occurs spontaneously in one third of the patients within 2–3 years [21, 56]. The recovery rate lies between 35% and 46% after 1.9 years [8] but it has been reported to be higher [19, 31]. In COVID-19-associated OD, the recovery rate is at 43% after an average of 3.5 months [47]. Recent studies have shown that the prevalence of long-term olfactory impairment lies between 2.9% and 8.3% 2 years after COVID-19 infection [3, 34].

The aim of this study was to investigate the demographic and clinical characteristics in a large group of patients with parosmia.

## Material and methods

### Study design

The present study is a retrospective data analysis of patient data, which were collected during consultations at the Smell and Taste Outpatient Clinic of the Department of Otorhinolaryngology at the University Hospital Dresden from February 2021 to July 2022.

The investigation was approved by the ethics committee of the medical faculty of the Technical University of Dresden (BO-EK-254062022). The study was designed and conducted in accordance with the ethical principles defined in the current revised version of the 2013 Declaration of Helsinki.

### Participants

Data were collected from 297 patients (203 female patients, 91 male patients, 3 not specified). Although there were isolated missing data, no patient was excluded as each variable was analyzed on a case-by-case basis. The patients were divided into two groups according to their medical history: parosmia present and parosmia not present. Only patients with PIOD (non-COVID), including COVID-19, were enrolled in the study. Therefore, a further distinction was made between COVID and non-COVID in the parosmia group. The differentiation between COVID and non-COVID was made on the basis of medical history and partly by presenting a polymerase chain reaction (PCR) test result. Other causes of OD, such as chronic rhinosinusitis, neurodegenerative diseases, or head trauma, were excluded.

### Methods

#### Clinical examination and parosmia questionnaire

As part of the routine presentation in our smell and taste consultation, each patient received a detailed medical history, especially regarding the presence of parosmia and phantosmia symptoms. According to the frequency (daily, not daily), intensity (very intense, less intense), and consequences (weight loss, change of daily activities) of the symptoms, the parosmia was graded from 0 to III degrees [23]. In addition, participants underwent nasal endoscopy by a certified otorhinolaryngologist to identify pathologies, such as massive septal deviations, nasal polyps, and signs of inflammation. To further evaluate the qualitative olfactory dysfunction some of the patients (*n* = 115) were asked to complete the parosmia questionnaire (PQ; Table 1), as used in a previous study by Landis and colleagues [33]. Each response was scored, and the summated score was the PQ score (min 4, max 16 points). A low PQ value is associated with a higher probability of occurrence of parosmia.

**Table 1:** Parosmia questionnaire including four questions in order to elicit the presence or absence of parosmia

Question	Possible answers	Points
Food tastes different from what it used to	I completely agree	1
	I mostly agree	2
	I mostly disagree	3
	I completely disagree	4
Often, I perceive a bad smell, regardless of whether a potential odor source is present	I completely agree	1
	I mostly agree	2
	I mostly disagree	3
	I completely disagree	4
Other people find odors pleasant which are unpleasant to me	I completely agree	1
	I mostly agree	2
	I mostly disagree	3
	I completely disagree	4
The biggest problem is not that odors are less intense (or absent), but that things smell different from what they used to	I completely agree	1
	I mostly agree	2
	I mostly disagree	3
	I completely disagree	4

Additionally, depending on the degree of parosmia, a subgroup analysis was performed for patients with higher or lower severity of the degree of parosmia (degrees < II and ≥ II).

#### “Sniffin’ sticks” and TDI score

Quantitative olfactory function was assessed using the “Sniffin’ Sticks” test battery (Burghart, Holm, Germany). Odor threshold (T), odor discrimination (D), and odor identification (I) scores were assessed as described previously [51]. The sum of the results yields the TDI score, on the basis of which the olfactory function can be divided into anosmia (TDI ≤ 16 points), hyposmia (16 < TDI ≤ 30.5 points), and normosmia (TDI ≥ 30.75 points).

#### Subjective olfactory function

The patients were asked either to mark their olfactory function on a visual analogue scale (VAS) of 10 cm length, the left end indicating no olfactory function and the right end perfect olfactory function, or to write down their olfactory function in percent. The selection of the VAS was converted into a score ranging from 0 to 100.

#### Statistical analysis

For data processing, the Microsoft Office 365 version 2017 database (Microsoft Corp., Redmond, WA, USA) was used. The graphs were created using Microsoft Office (Microsoft Corp.) and OriginPro (OriginLab Corp., Northampton, MA, USA). Statistical analysis was performed using SPSS (Statistical Package for the Social Sciences, version 29; SPSS Inc., IBM Corp., Chicago, IL, USA). Prior to statistical evaluation, the data were analyzed with respect to normal distribution through parametric and nonparametric tests were performed. A significance level of *p* < 0.05 was set. Results are reported as mean (± standard deviation, SD) unless declared otherwise.

## Results

**Fig. 1:**
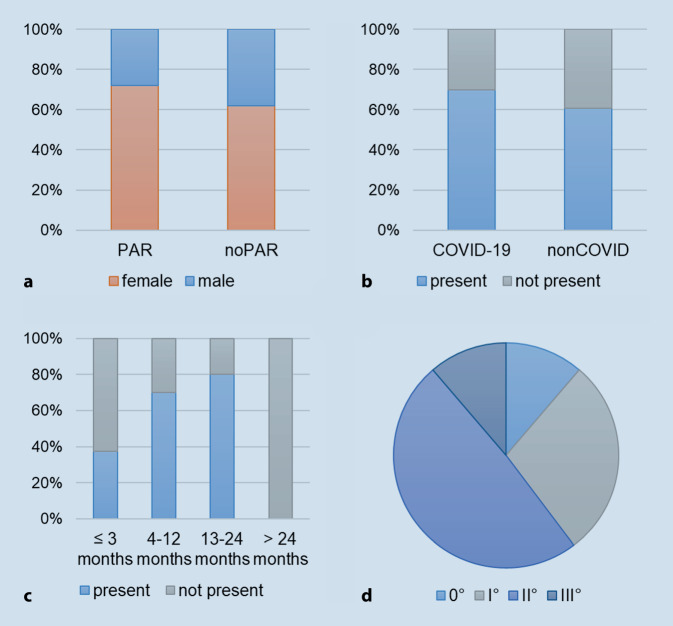
**a** Gender ratio in the groups with parosmia present and not present. **b** Proportion of parosmia in the COVID-19 and non-COVID groups. **c** Ratio of duration of disease in the groups with parosmia present and not present subdivided into ≤ 3 months, 4–12 months, 13–24 months, and > 24 months. **d** Distribution of parosmia degree in the study population

In total, the information of 297 patients was included in this study (203 female patients, 91 male patients, 3 not specified). The age of the patients with parosmia (PAR; *n* = 201, 69%) was significantly younger compared to the group without parosmia (noPAR; *n* = 92, 31%; PAR 43 ± 13 years, noPAR 47 ± 15 years; *p* = 0.03). More than two thirds of the study population (*n* = 204) had PAR, of whom 144 were female patients vs. 57 in the noPAR group (*p* = 0.097). There was no difference between the COVID-19 and non-COVID group in the proportion of patients with parosmia (COVID-19, 70% vs. non-COVID, 61%, *p* = 0.23; Fig. 1). Overall, 60 patients of the study population reported phantosmia and the majority of these were post-COVID cases (*n* = 53, 88%). Of these patients, 35 also complained of parosmia (*n* = 35, 61%). Regarding the mean duration of the OD, there was no statistically significant difference between the two groups (PAR 10.3 ± 4.9 months, noPAR 13.6 ± 37.6 months, *p* = 0.21, 1–344 months).

The severity of the qualitative disorder was graded from 0 to III degrees in 204 patients. In total, 23, 58, 100, and 23 patients were diagnosed with parosmia of degrees 0, I, II, and III, respectively. The subgroup analysis for parosmia degree < II and ≥ II showed no significant differences between the two groups with respect to age, gender, or duration of disease.

The self-rated olfactory function, given in percent (%) by the patients (*n* = 185), was significantly better in patients with parosmia (*n* = 125; PAR 33.7 ± 22%, noPAR 22.8 ± 17.7%, *p* = 0.001). The TDI score was collected from a total of 295 patients, of whom 202 had parosmia. The mean TDI score (PAR 24.3 ± 7.0 points, noPAR 21.4 ± 8.2 points, *p* = 0.003), T score (PAR 4 ± 2.8 points, noPAR 3.3 ± 2.6 points, *p* = 0.043), and D score (PAR 10.4 ± 3 points, noPAR 9.6 ± 3.4 points, *p* = 0.04) were significantly better in patients with parosmia than without. This did not apply to the I score (PAR 9.7 ± 3.1 points, noPAR 9.1 ± 3.5 points, *p* = 0.17). There was a significant correlation between subjective olfactory function and the TDI score in our study population (Pearson *r* = 0.57, *p* < 0.001). This also applied to the T (Pearson *r* = 0.55, *p* < 0.001) and I score (Pearson *r* = 0.48, *p* < 0.001), but not to D score (Pearson *r* = −0.10, *p* = 0.17). Regarding quantitative olfactory function (T, D, I, and TDI score), there were no statistically significant differences in the subgroup analysis by parosmia degree as shown in Table 2.

**Table 2:** Demographic information and results of the olfactory testing^a^

	Study population (*n* = 297)	Parosmia present (*n* = 201)	Parosmia not present (*n* = 92)	*p*	Parosmia < II° (*n* = 81)	Parosmia ≥ II° (*n* = 123)	*p*
*Age*	44 (14)	43 (13)	47 (15)	0.03	44 (12)	42 (14)	0.414
*Cause *(%)							
COVID	250 (84.2)	174 (69.9)	75 (30.1)	0.234	70 (39.8)	106 (60.2)	0.567
non-COVID	47 (15.8)	28 (60.9)	17 (39.1)		11 (39.3)	17 (60.7)	
*Gender* (%)							
♀	203 (68.4)	144 (72)	57 (62)	0.103	59 (73.8)	85 (69.7)	0.634
♂	91 (30.6)	58 (28)	35 (38)		21 (26.2)	37 (30.3)	
*Duration of disease *(months)	11.4 (21.1)	10.3 (4.9)	13.6 (37.6)	0.231	10 (4.6)	10.5 (5)	0.479
*T score*	3.8 (2.7)	4 (2.8)	3.3 (2.6)	0.043*	4.3 (2.7)	3.8 (2.8)	0.227
*D score*	10.1 (3.1)	10.4 (3)	9.6 (3.4)	0.04*	10.7 (2.8)	10.2 (3.2)	0.2
*I score*	9.5 (3.2)	9.7 (3.1)	9.1 (3.5)	0.169	9.7 (2.8)	9.7 (3.2)	0.883
*TDI score*	23.4 (7.5)	24.3 (7)	21.4 (8.2)	0.003*	24.6 (6.8)	24.2 (7.1)	0.676
*PQ question 1*	2 (0.9)	1.8 (0.8)	2.4 (1.1)	0.002*	2 (0.9)	1.7 (0.8)	0.127
*PQ question 2*	3.2 (0.9)	3.1 (0.9)	3.5 (0.7)	0.008*	3.4 (0.7)	2.9 (1)	0.008*
*PQ question 3*	2.7 (1.1)	2.2 (1.1)	3.6 (0.5)	< 0.001*	2.8 (1)	2 (1)	0.001*
*PQ question 4*	2.3 (1.1)	1.7 (0.9)	3.4 (0.7)	< 0.001*	2.3 (1)	1.5 (0.7)	< 0.001*
*PQ score*	10.2 (3.1)	8.8 (2.6)	13 (1.9)	< 0.001*	10.6 (2.5)	7.5 (1.9)	< 0.001*

^a^Results are shown as mean (± standard deviation) if not stated differently

*Statistically significant

**Fig. 2:**
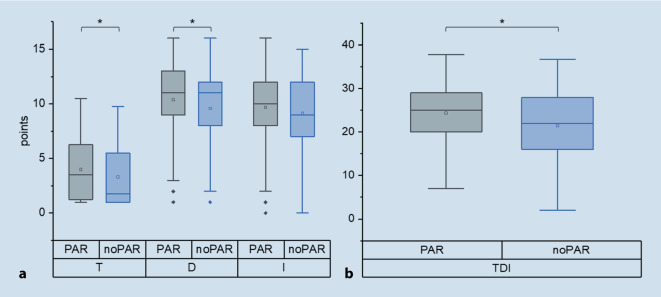
Box plot of odor threshold (*T*), discrimination (*D*), identification (*I*), and TDI scores (*TDI*) in patients with parosmia (PAR) and without (noPAR). *Statistically significant differences between the groups; □ median value, ♦ outliers

The analysis of the PQ showed that there were statistically significant differences for all four que stions of the PQ as well as the PQ score between the PAR (*n* = 78) and the noPAR (*n* = 35) group (question 1, PAR 1.8 ± 0.8, noPAR 2.4 ± 1.1, *p* = 0.002; question 2, PAR 3.1 ± 0.9, noPAR 3.5 ± 0.7, *p* = 0.008; question 3, PAR 2.2 ± 1.1, noPAR 3.6 ± 0.5, *p* < 0.001; question 4, PAR 1.7 ± 0.9, noPAR 3.4 ± 0.7, *p* < 0.001; PQ score, PAR 8.8 ± 2.6, noPAR 13 ± 1.9, *p* < 0.001; Fig. 2).

We also observed a negative correlation between the degree of parosmia and the PQ score in the group of patients with parosmia (Pearson *r* = −0.59, *p* < 0.001). Furthermore, the subgroup analysis showed a significant difference in the PQ score between parosmia degrees < II and ≥ II. However, there was no significant correlation between the PQ score and the parosmia degree when analyzing the two groups separately (PAR < II Pearson *r* = −0.38, *p* = 0.07; PAR ≥ II Pearson *r* = −0.18, *p* = 0.20).

## Discussion

Patients affected by parosmia were younger than patients without parosmia and had a better olfactory function. These findings also match the results of an online survey conducted by Pellegrino and colleagues [54] but not those of Menzel et al. because we could not show a difference in gender and duration of disease when parosmia is present or not [47].

Several studies suggest that there is a discrepancy between the self-rated olfactory function and olfactory test results [32, 43, 61, 62]. While self-ratings and self-descriptions of olfactory function are of the highest significance in clinical olfaction, a structured history in a personal interview and the clinical examination are equally important in acquiring a less biased view of the complaints. Accordingly, olfactory tests are inevitable for assessing olfactory function. Still, there was a correlation between self-rated olfactory function and the TDI, T, and I score in our study population.

Compared to PIOD from the pre-COVID era, the proportion of individuals with parosmia appeared to have increased since COVID-19. In our study population, more than two thirds of the patients complained of parosmia (PAR 69% vs. noPAR 31%). In other studies, the prevalence was up to 76% [47, 50, 60]. However, in the pre-COVID-19 pandemic period it was between 18% and 56% depending on the study population [30, 49, 56, 57, 67]. For the last 2 years, mainly patients with post-COVID OD presented for consultation, which explains the unbalanced ratio of non-COVID and COVID-19 patients in our study cohort, as well as the longer duration of disease for the non-COVID group. In addition, only outpatients were included in our study, and therefore no general conclusion can be drawn regarding overall olfactory dysfunction in COVID-19. Furthermore, it should be noted that within our observation period there were different SARS-CoV‑2 variants (peak of alpha: April–May 2021, peak of delta: September–November 2021, peak of omicron: December 2021–February 2022; [15]).

To date, there is no validated test available for the diagnosis of parosmia; only structured medical history and the parosmia questionnaire of Landis et al. are used [33]. Several clinical findings were associated with parosmia such as a reduced volume of the olfactory bulb [48, 57, 58] and specific patterns in central processing of olfactory stimuli in functional magnetic resonance imaging (fMRI; [26]). However, none is suited for clinical diagnostics at an individual level. With our study, we confirmed that the PQ is suitable for distinguishing between patients with parosmia and patients without parosmia. We also observed a negative correlation between the degree of parosmia and the PQ score in the PAR group. Furthermore, the subgroup analysis showed a significant difference in the PQ score between parosmia degrees < II and ≥ II. However, there was no significant correlation between the PQ score and the parosmia degree in these groups, implying that the PQ is suitable for screening for the presence of parosmia but not for grading. Furthermore, the degree of parosmia should be evaluated depending on the symptoms [23]. Liu et al. proposed the “SSParoT” based on the hedonic rating of odors as a tool to assess qualitative OD in healthy and affected individuals [41]. However, the discrimination between parosmia and non-parosmia seems to be problematic [60].

Treatment options for parosmia are limited. Recently Altundag et al. demonstrated a positive effect of modified olfactory training in patients with parosmia following a SARS-CoV‑2 infection [1]. Another study revealed that the presence of parosmia is associated with a relevant recovery in olfactory function in PIOD patients after olfactory training [40]. Therefore, olfactory training seems to be an effective therapy for parosmia. Garcia and colleagues treated 12 post-COVID patients with gabapentin in addition to olfactory training and topical corticosteroids [18]. After 3 weeks of treatment a significant improvement was noticed in 67% of the patients. So far, gabapentin has also been shown to have a positive benefit for phantosmia in isolated cases [10]. Only few medical treatment options for parosmia have been proposed in the past. Zilstorff applied cocaine hydrochloride to the olfactory mucosa that led to temporary blockage of olfactory distortion due to an anesthetic effect by blocking of the sodium channel and interfering with action potential [69]. Oral substitution of alpha-lipoic acid per day for an average of 4.5 months resulted in a reduction in the percentage of parosmia in patients with post-infectious olfactory disorders (48% before vs. 22% after treatment; [22]). Although a beneficial effect of the treatment with alpha-lipoic acid was reported, no further investigations have been published since then. Intranasal application of sodium citrate led to a reduction in the proportion of patients with parosmia after treatment (38% before vs. 25% after treatment) but without reaching statistical significance (*p* = 0.17) [67]. The surgical resection of olfactory mucosa is a more invasive but possibly beneficial approach in severe qualitative OD [27, 38, 45]. To date there is one study proposing a successful treatment of unilateral peripheral parosmia by olfactory cleft blockage [42].

Parosmia is considered a positive prognostic predictor for olfactory recovery, especially in patients with PIOD [24, 40, 47, 56]. However, this was not confirmed in other studies [55]. Still, it seems to be of high importance to inform the patients about this positive aspect, because parosmia is occurring more frequently since COVID-19, and some patients do not know how to cope with this symptom. It gives hope to the patients with parosmia, which is important, because of the association between parosmia and depression and/or anxiety [13, 35].

## Conclusion

Patients affected by parosmia were younger and had a better olfactory function compared to patients without parosmia. Qualitative olfactory dysfunction can have a major impact on the patients’ quality of life, and therefore identifying these patients is mandatory in order to provide appropriate therapy. This requires further research due to the lack of specific therapy options for parosmia.

## Declarations

All procedures performed in studies involving human participants or on human tissue were in accordance with the ethical standards of the institutional and/or national research committee and with the 1975 Helsinki declaration and its later amendments or comparable ethical standards. The investigation was approved by the ethics committee of the medical faculty of the Technical University of Dresden (BO-EK-254062022). Informed consent was obtained from all individual participants included in the study.
